# Electron microscopic characteristics of interstitial cystitis/bladder pain syndrome and their association with clinical condition

**DOI:** 10.1371/journal.pone.0198816

**Published:** 2018-06-07

**Authors:** Jia-Fong Jhang, Han-Chen Ho, Yuan-Hong Jiang, Cheng-Ling Lee, Yuan-Hsiang Hsu, Hann-Chorng Kuo

**Affiliations:** 1 Department of Urology, Buddhist Tzu Chi General Hospital and Tzu Chi University, Hualien, Taiwan; 2 Department of Anatomy, Tzu Chi University, Hualien, Taiwan; 3 Department of Pathology, Buddhist Tzu Chi General Hospital and Tzu Chi University, Hualien, Taiwan; University of Oklahoma Health Sciences Center, UNITED STATES

## Abstract

**Background:**

Electron microscopy (EM) characteristics of the urothelium in interstitial cystitis/bladder pain syndrome (IC/BPS) and their association with clinical condition are unclear.

**Methods:**

Ten IC/BPS patients who were admitted for hydrodistention and 5 patients with stress urinary incontinence (control patients) were enrolled. All patients provided detailed clinical histories and underwent urodynamic studies. Cystoscopic bladder biopsies were obtained and processed for transmission EM (TEM) and scanning EM (SEM). The severity of the urothelium findings was graded on a 4-point scale (0: none, 1: mild, 2: moderate, and 3: severe). The EM findings between IC/BPS and control patients were compared; the results were analyzed using the chi-square test.

**Results:**

Compared with the urothelium of control patients, the urothelium of IC/BPS patients had more severe defects of the urothelial cell layers and integrity of umbrella cells in TEM (p = 0.045 and 0.01, respectively). In SEM, umbrella cell pleomorphism increased and microplicae of the cell membrane decreased in the IC/BPS group, and both were more severe than in the control group (p = 0.022 and 0.007, respectively). The patients with moderate to severe defects of umbrella cell integrity had more severe bladder pain and smaller maximal bladder capacity (MBC) (both p = 0.010). Patients with moderate to severe defects in microplicae of the cell membrane had smaller cystometric bladder capacity and MBC (p = 0.037 and 0.047, respectively).

**Conclusions:**

The results revealed significant urothelium defects in IC/BPS, especially in the umbrella cells. Defects of umbrella cells may play an important role in the pathogenesis of IC/BPS.

## Introduction

Interstitial Cystitis/Bladder Pain Syndrome (IC/BPS), which is characterized by bladder pain and lower urinary tract symptoms, is a tricky urological disease to diagnose and treat [1]. IC/BPS is more common in women than in men, and according to high sensitivity and specificity definitions, the estimated prevalence of IC/BPS in women was 6.5% and 2.7%, respectively. [2,3] Over the past 30 years, investigators have focused significant effort aimed at understanding the pathogenesis of this disease, but the detail mechanisms remain a mystery [1]. Recently, the role of urothelial dysfunction in the pathogenesis of IC/BPS has attracted considerable interest [4]. Previous studies revealed defects of surface glycosaminoglycan and increased epithelial permeability in the urothelium of IC/BPS patients [5,6]. Immunochemical staining study also found the cell tight junction protein zonula occludens-1 and adhesive junction protein E-cadherin were significantly decreased in the bladders of patients with IC/BPS [7]. Furthermore, up-regulation of the purinoceptor P2X3 and increasing adenosine triphosphate release in the urothelium are also reported in the patients with IC/BPS [8,9]. Currently, dysfunction of the urothelium is considered a key pathological mechanism of IC/BPS.

Electron microscopy (EM) has been used to investigate the ultrastructure of bladder epithelial cells in patients with IC/BPS for the past 40 years [10]. Defects of junctional complexes, epithelial pleomorphism, microvilli, and mast cell activation in IC/BPS bladders have been previously described in several EM studies[11–13]. However, some EM studies found no differences in the morphologic appearance of the urothelium in IC/BPS patients when compared with controls [14,15]. Although histological and immunochemical staining studies have revealed urothelial defects in IC/BPS, the abnormalities in the human urothelium remain controversial. The correlation between urothelial defects on EM and the clinical symptoms of IC/BPS is also unclear. Therefore, the aim of the current study is to investigate the urothelial morphologic characteristics using transmission EM (TEM) and scanning EM (SEM) in patients with ulcerous and non-ulcerous IC/BPS and to correlate the EM findings with clinical symptom severity.

## Methods

### Patient selection

From July 2016 to April 2017, patients with IC/BPS who were admitted to our hospital for diagnostic cystoscopic hydrodistention were prospectively enrolled in the study. The diagnosis of IC/BPS was made according to the European Society for the Study of Interstitial Cystitis criteria in 2008 (chronic bladder pain or discomfort accompanied by at least one other urinary symptom such as urinary frequency or urgency).[1] Patients with concurrent urological diseases such as acute or chronic bacterial cystitis, urolithiasis, ketamine-related cystitis, or neurogenic voiding dysfunction were excluded. The patients who had undergone urological procedures within the previous 6 months, such as cystoscopic hydrodistention or intravesical instillation of any therapeutic agent, were also excluded.

The institutional review board and ethics committee of Buddhist Tzu Chi General Hospital approved this study (IRB number 105-28-A). Each participant provided written informed consent. In addition, patients with stress urinary incontinence who were admitted to the hospital for anti-incontinence surgery were offered enrollment as control patients. All of the IC/BPS patients and the control patients are female.

### Clinical evaluation

All patients underwent comprehensive medical history reviews after admission to the hospital. The bladder pain was evaluated with Visual Analog Scale (VAS) for pain, the patients were asked to rate their bladder pain on a scale of 1–10. [16] All patients also underwent urodynamic studies to confirm the diagnosis of IC/BPS and rule out the co-existence of other lower urinary tract diseases such as bladder outlet obstruction. The cystometric bladder capacity (CBC) was recorded. After admission, all patients underwent cystoscopic hydrodistention under general anesthesia, and the patients were classified as having ulcerous or non-ulcerous IC/BPS according to the cystoscopic findings of Hunner’s lesion before the bladder distention. The hydrodistention was performed at an intravesical pressure of 80 cm of water, and the maximal bladder capacity (MBC) were recored. Following hydrodistention, cold-cup biopsies were obtained from non-bleeding sites in the posterior bladder wall. In 3 of the patients, bladder biopsies were also obtained before the hydrodistention to evaluate the impact of this procedure on the urothelium. Each specimen was 2 mm in diameter and contained mucosal and submucosal tissues. Endoscopic electrocauterization of the biopsy sites was done to prevent bladder bleeding. Bladder biopsies in control patients were also similarly obtained and prepared for EM investigation.

### Transmission electron microscopy

The bladder biopsy specimens were immediately washed 3 times in cold buffer and prefixed with 2.5% glutaraldehyde/0.1 M cacodylate buffer (pH 7.3) at 4°C for at least 1 hour, followed by post-fixation with 1% osmium tetroxide/0.1 M cacodylate buffer for 1 h at room temperature. After staining with 2% aqueous uranyl acetate, specimens were dehydrated and embedded in Spurr’s resin. Ultrathin sections of 70–80 nm were cut on a Leica Ultracut R ultramicrotome, collected on formvar-coated single slot grids, and examined under a Hitachi H-7500 transmission electron microscope (Hitachi, Tokyo, Japan) at 80 kV. The urothelium cell layer numbers, the integrity of umbrella cells, and anchoring junctions were investigated and graded using a 4-point scale (0: normal, 1: mild defect, 2: moderate defect, 3: severe defect) for both the IC/BPS and control groups.

### Scanning electron microscopy

The bladder biopsy specimens were prepared and fixed with glutaraldehyde and osmium tetroxide as described above. The specimens were then dehydrated through a graded series of ethanol till 100% ethanol, and replaced with 100% acetone. The specimens were critical point dried and sputter coated with gold. Specimens were examined with a Hitachi S-4700 field emission scanning electron microscope at 15 kV. For SEM in both groups, the umbrella cell size and microplicae of the cell membrane were observed and a 4-point scale was used for grading. All of the EM findings were graded by a single investigator who was masked to the clinical results.

### Statistical analysis

The chi-square test was used to analyze the significance of the differences in EM characteristic grading between the IC/BPS and control groups. The EM findings between ulcerous and non-ulcerous IC/BPS was also analyzed using the chi-square test. The patients with IC/BPS patients were classified into two groups according to the severity of urothelial defects on TEM and SEM (none and mild vs. moderate and severe). Differences in the quantitative symptom parameters (VAS, CBC, and MBC) between the EM finding groups were compared using the Mann–Whitney U test. P-values less than 0.05 were considered significant. All calculations were performed using SPSS for Windows, version 16.0 (SPSS, Chicago, IL).

## Results

Ten patients with IC/BPS (mean age, 56.6 ± 7.3 years, range from 33 to 75 years) and 5 control patients (mean age, 64.4 ± 9.0 years, range from 58–79 years) were enrolled in the study. The mean VAS pain score in the IC/BPS group was 3.5 ± 2.7; the CBC was 235.0 ± 110.0 mL, and the MBC was 600 ± 147.2 mL. Of the 10 patients in the IC/BPS group, 3 had ulcerous IC/BPS, and the remaining 7 had non-ulcerous IC/BPS. Five patients with non-ulcerous IC/BPS had grade 2 glomerulation during cystoscopic hydrodistention while the other 2 patients had grade 3 glomerulation.

**Fig 1:**
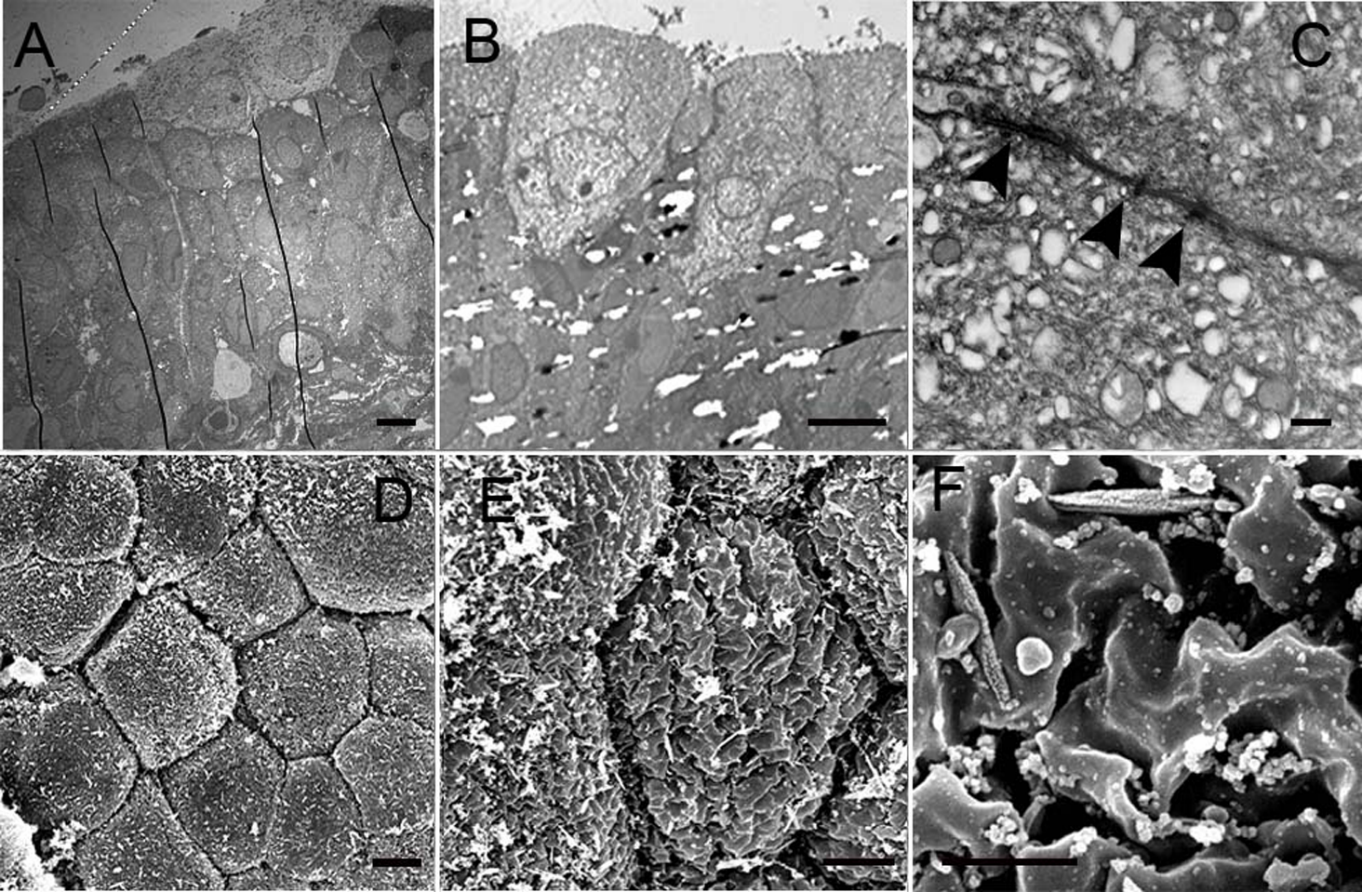
TEM of control specimens. TEM of control specimens revealed 3–6 cell layers in the urothelium (A, bar: 10um) with intact umbrella cells (B, bar: 10um) and anchoring junctions such as desomosomes. (C, bar:500nm). SEM also revealed intact umbrella cells with uniform cell size (D, bar: 10um). The microplicae in the umbrella cells was also intact (E, bar: 5um and F, bar: 1um).

**Fig 2:**
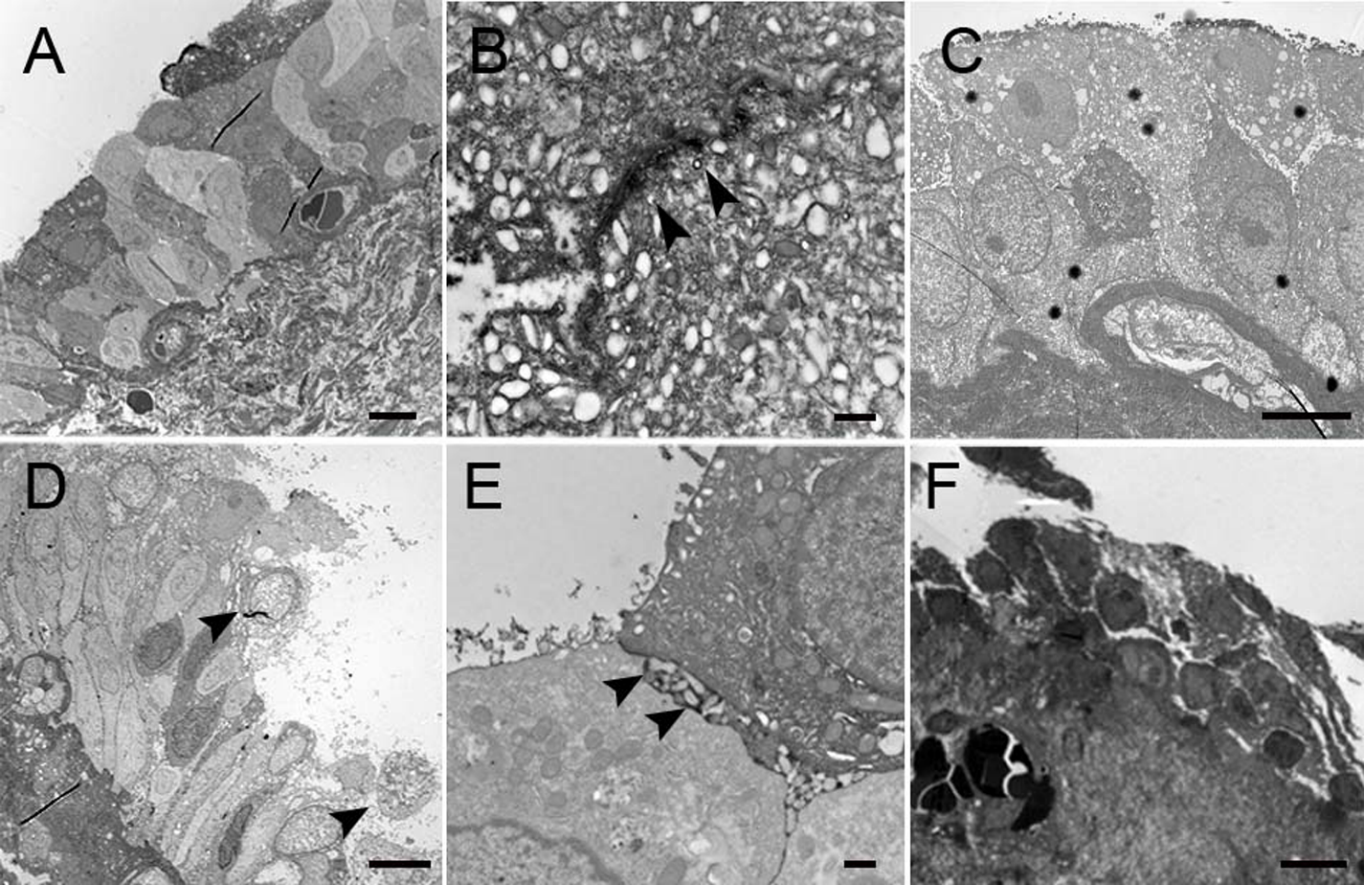
TEM for IC/BSP bladder samples. TEM of IC/BPS samples from patients with different symptom severity showed variable findings. In some IC/BPS patients, normal cell layers in the urothelium and intact, anchoring junctions were found (A, bar: 10um and B, bar:500nm). However, most IC/BPS patients had urothelial defects on TEM. Decreased cell layers (C, bar: 10um), denudation of umbrella cells (D, arrowheads, bar: 10um), and widening of lateral interstitial space between adjacent umbrella cells were noted in most patients (E, bar:500nm). In patients with severe ulcerous IC/BPS, only a few basal cells could be found in the urothelium (F, bar:10um).

**Fig 3:**
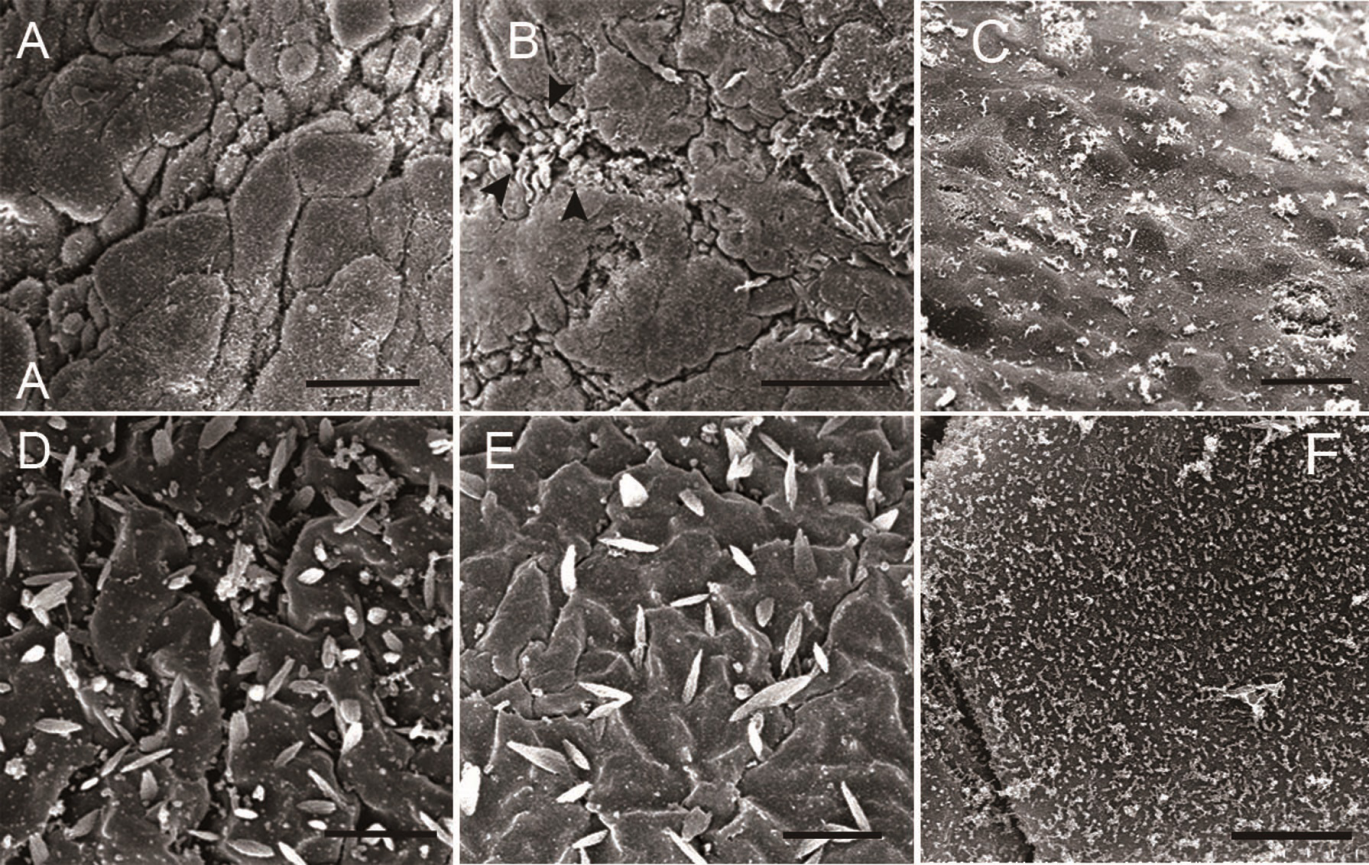
SEM for IC/BPS bladder samples. SEM of IC/BPS specimens showed umbrella cell pleomorphism (A, bar: 40um) and denudation with intermediate cell exposure (B, arrowhead, bar: 100um). In some severe IC/BPS, totally denudation of urothelium with exposed basement membrane was observed (C,bar 20um). In some patients, microplicae of the umbrella cell membranes was only mild decreased (D, bar: 2um), but most patients had moderate to severe decreases of microplicae (E, bar: 2um and F, bar: 5um).

Figs 1–3 show representative TEM and SEM images of IC/BPS and control urothelium. In control samples, TEM revealed that the urothelium consisted of 3–6 cell layers with intact umbrella cells and anchoring junctions (Fig 1A–1C). The SEM also showed intact uniform umbrella cells with deep microplicae in the cellular surface (Fig 1D–1F). However, some urothelial defects were also observed in control subjects. Table 1 provides the urothelial defects and grading results and clinical parameters in the IC/BPS and control groups. In IC/BPS specimens, TEM revealed decreased urothelium cell layers with umbrella cell loss (Fig 2C and 2D). Lateral interstitial space between adjacent umbrella cells was also widening in most IC/BPS samples (Fig 2E). SEM showed umbrella cell pleomorphism and denudation (Fig 3A and 3B). In some severe cases of IC/BPS, severe urothelium denudation with exposed basement menbrane was observed (Fig 3C). The microplicae in the umbrella cells was also decreased (Fig 3D–3F). Table 2 shows a comparison of urothelial defect detected under EM. In TEM, the chi-square test revealed more severe defects of the urothelium cell layers and defects in the integrity of the umbrella cells in the IC/BPS group than in the control group (p = 0.045 and 0.010, respectively). In SEM, the IC/BPS group also had more severe umbrella cell pleomorphism and defects of microplicae of the cell membrane (p = 0.022 and 0.007, respectively). Three of the patients had bladder biopsies before and after hydrodistention. The EM findings did not differ between the 2 biopsies.

**Table 1:** Summary of clinical parameters and grading of electron microscopy (EM) findings in IC/BPS and control patients.

IC/BPS Patients								
No.	VAS	CBC (mL)	MBC (mL)	TEM Findings			SEM Findings	
				Urothelial cell layers	Umbrella cell integrity	anchoring junction	Umbrella cell pleomorphism	microplicae
1	0	400	800	2	1	2	2	1
2	0	150	700	1	1	2	2	3
3 (ulcer)	5	217	400	3	3	3	3	3
4	3	241	600	3	3	3	2	2
5	4	178	500	3	3	3	3	3
6	7	201	500	3	3	3	2	3
7	2	393	750	1	1	1	1	1
8 (ulcer)	7	100	600	3	3	3	3	3
9 (ulcer)	6	120	400	1	2	2	1	2
10	1	350	750	0	1	0	1	2
**Controls subjects**								
1	N/A	N/A	N/A	0	0	0	0	0
2				0	0	2	0	1
3				0	1	1	1	0
4				1	0	2	0	0
5				0	0	0	1	0

EM grading: 0 = normal, 1 = mild defect, 2 = moderate defect, 3 = severe defect. VAS: Visual Analogue Scale pain score; CBC: cystometric bladder capacity, MBC: maximal bladder capacity.

**Table 2:** Comparison of urothelium EM defect findings between IC/BPS and control patients.

	IC/BPS Patients (n = 10)				Control Patients (n = 5)				P-value
**TEM Findings**	None	Mild	Moderate	Severe	None	Mild	Moderate	Severe	
Urothelial cell layers	1 (10%)	3 (30%)	1 (10%)	5 (50%)	4 (80%)	1 (20%)	0 (0%)	0 (0%)	0.045
Umbrella cell integrity	0 (0%)	4 (40%)	1 (10%)	5 (50%)	4 (80%)	1 (20%)	0 (0%)	0 (0%)	0.010
anchoring junction	1 (20%)	1 (20%)	3 (10%)	5 (50%)	2 (40%)	1 (20%)	2 (40%)	0	0.226
**SEM Findings**	None	Mild	Moderate	severe	None	Mild	Moderate	Severe	
Umbrella cell pleomorphism	0 (0%)	3 (30%)	4 (40%)	3 (30%)	3 (60%)	2 (40%)	0 (0%)	0 (0%)	0.022
Microplicae	0 (0%)	2 (20%)	3 (30%)	5 (50%)	4 (80%)	1 (20%)	0 (0%)	0 (0%)	0.007

**Fig 4:**
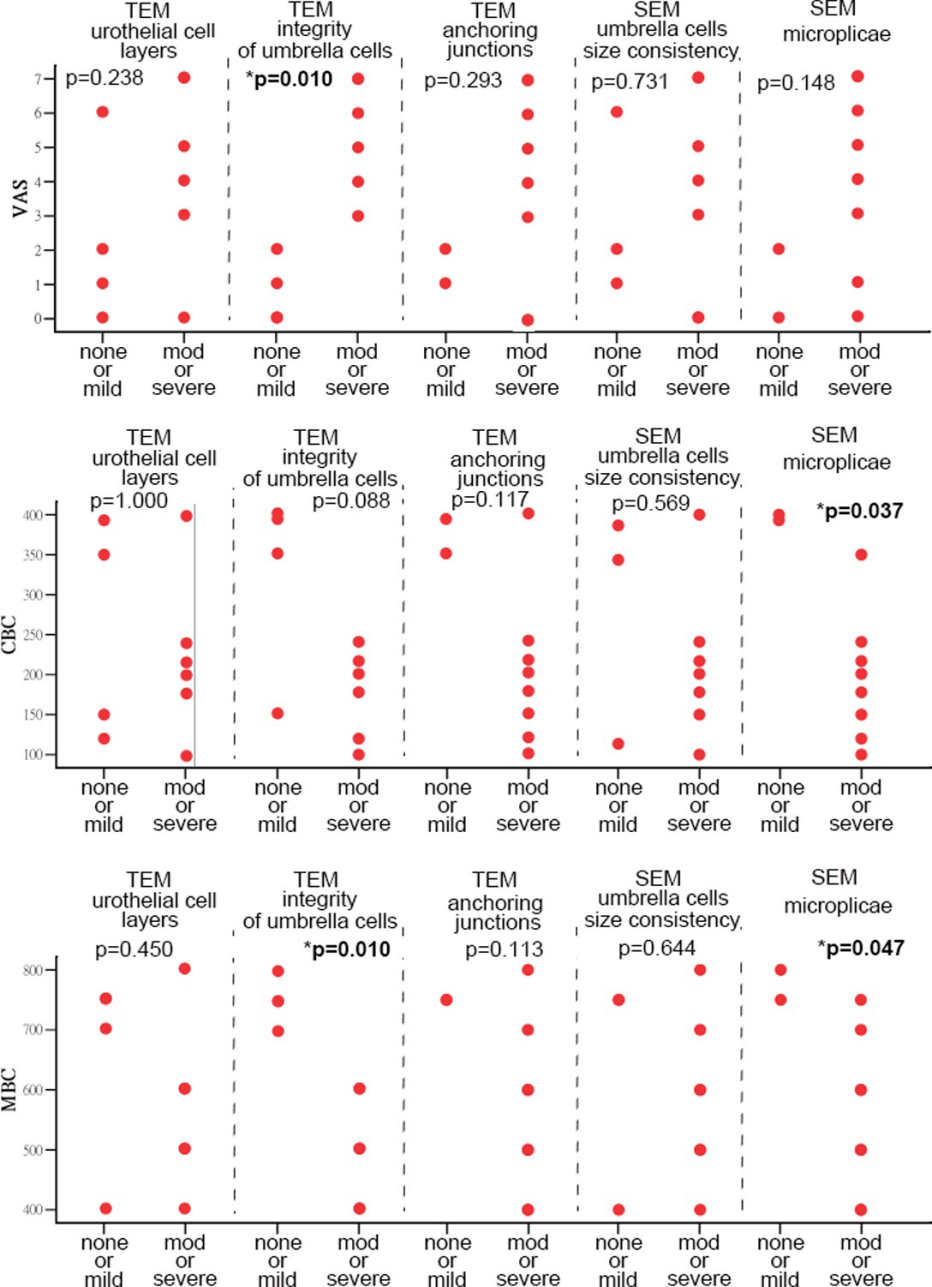
IC/BPS symptom parameters for the various EM finding severities. The patients with moderate to severe defects of umbrella cell integrity on TEM had significantly more severe VAS pain scores and smaller MBCs (both p = 0.010, Mann–Whitney U test). Patients with moderate to severe defects of microplicae in the cell membrane on SEM had significantly smaller CBCs and MBCs (p = 0.037 and 0.047, respectively, Mann–Whitney U test).

Fig 4 shows the IC/BPS symptom parameters by the severity of EM findings, including VAS, CBC, and MBC. On TEM, the patients with moderate to severe defects of umbrella cell integrity had significantly more severe VAS pain scores and smaller MBC (both p = 0.010). On SEM, patients with moderate to severe defects in the microplicae of the cell membrane had significantly smaller CBC and MBC (p = 0.037 and 0.047, respectively). In all 3 patients with ulcerous IC/BPS, severe urothelium defects were observed on TEM and SEM. Microvilli in the umbrella cells were found in all 3 ulcerous IC/BPS samples and 3 of 7 non-ulcerous IC/BPS samples. However, statistical results did not differ significantly between ulcerous and non-ulcerous IC/BPS.

**Fig 5:**
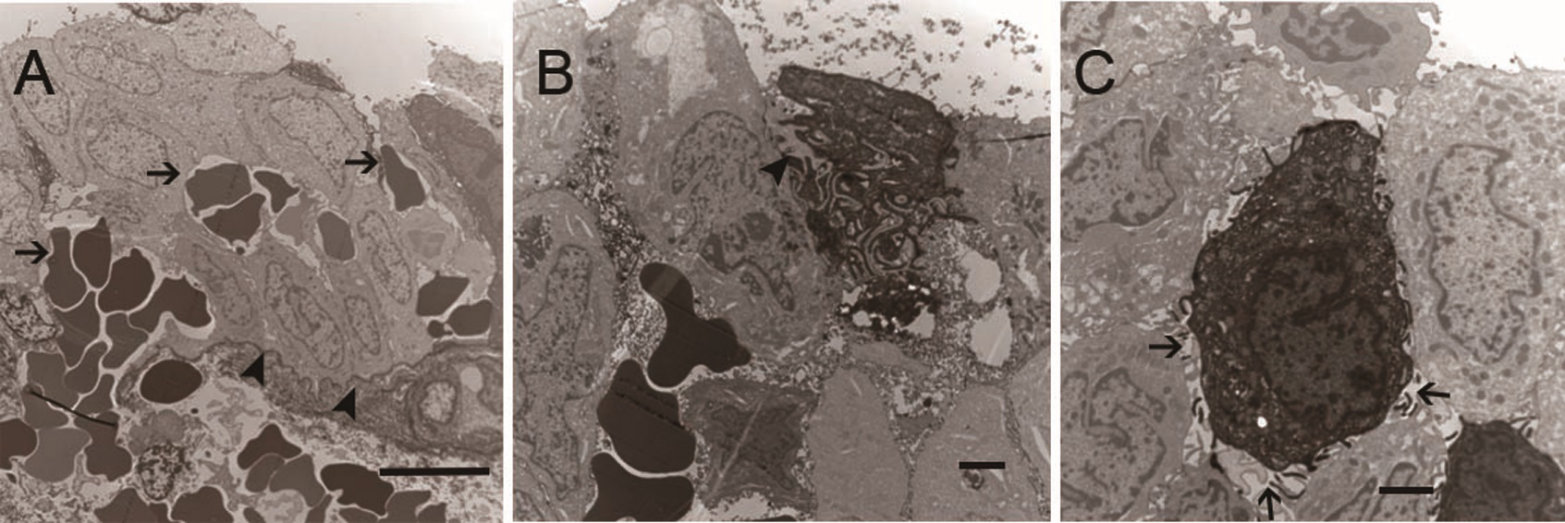
Novel TEM findings in IC/BPS urothelium. (A) red blood cells extravasation (black arrows) from lamina propria to urothelium with interrupted basement membrane (arrowheads, bar: 10um). This bladder specimen was obtained before hydrodistention. (B) Macrophage in the urothelium (arrowhead, bar: 2um). (C) Widening of lateral interstitial space between adjacent umbrella cells s with loose interdigitation (black arrows, bar: 2um) in both umbrella and intermediate cells.

Several novel TEM findings were also observed in IC/BPS urothelium samples but not in control samples. Red blood cell extravasation from vessels in the lamina propria to the urothelium with interrupted basement membrane was found in 4 of 10 patients (Fig 5A). This finding was also noted in the bladder specimens taken before hydrodistention. Macrophages were observed in the urothelium samples of 2 patients (Fig 5B). Lateral interstitial space between adjacent cells widening with loose interdigitation of both umbrella and intermediate cells were noted (Fig 5C).

## Discussion

The use of EM to investigate the ultrastructure of human IC/BPS bladder samples started 40 years ago [10]. Several urothelial abnormalities have been observed in different EM studies, including loss of tight junctions, umbrella cell pleomorphism, and microvilli [11–13]. However, whether or not the urothelium differs on EM between IC/BPS samples and control samples remains controversial [14,15]. We used EM to inspect the IC/BPS urothelium and compared these findings to those of a control group. Although some EM defects in the urothelium were also found in the control samples, the severity of the defects was greater in the IC/BPS group. Furthermore, we found the severity of the urothelial defects on EM was associated with clinical symptoms in these patients.

Although urothelial dysfunction is considered an important mechanism of IC/BPS, the urothelial histopathological aberrations in IC/BPS, especially in the non-ulcerous type, is still controversial. A recent histopathology study semiquantitatively measured the urothelium in patients with IC/BPS and found the remnant urothelium was significantly decreased in ulcerous IC/BPS [17]. However, only minimal urothelial denudation was noted in non-ulcerous IC/BPS. In current human EM study, we found significantly decreased urothelial cell layers and loss of umbrella cells in patients with IC/BPS. These findings not only showed EM morphological evidence of urothelial denudation in IC/BPS but also indicated the aberration might result from loss of umbrella cells.

We also found that patients with IC/BPS with moderate to severe loss of umbrella cells had more severe VAS pain scores. Loss of umbrella cells results in a defect of the bladder barrier function, leading to proteins or ionic substances gaining access to the suburothelial tissue, and may directly induce bladder pain in patients with IC/BPS.[18] Decreased microplicae in the umbrella cell membrane was associated with decreased CBC and MBC in our patients. The microplicae or ridges in the umbrella cell membrane become flattened during distension and have an important role in bladder physiology [19]. Decreased microplicae of the umbrella cell membrane is believed to be associated with immature umbrella cells and may debilitate bladder distention. In addition, umbrella cell pleomorphism was also noted on SEM, which also suggests immaturity of the umbrella cells [19].

Glomerulation hemorrhage after hydrodistention is a common cystoscopy finding and is considered a diagnostic marker of IC/BPS [1]. We found red blood cell extravasation and breakage of the basement membrane in bladder specimens without any gross bleeding or petechia on TEM. This finding also occurred in the samples observed before hydrodistention. Previously, the role of bladder microvascular injury in IC/BPS attracted little discussion. A previous immunochemical study revealed increased vascular endothelial growth factor (VEGF) expression in IC/BPS bladder specimens [20]. Upregulation of VEGF may induce immature angiogenesis, in which microvessels have insufficient coverage of pericytes, resulting in hemorrhagic vessels [21]. According to our results, the microvascular injury might be a pathologic feature of IC/BPS urothelium, and may lead to glomerulation hemorrhage after hydrodistention.

The main limitations of this study are its small sample size and the lack of quantitative analyses. In such a small sample, the data could be heavily weighted by a few extreme values. Additionally, a single investigator grading the bladder specimens may lead to subjective bias in interpretation. The effect of cold-cup biopsy on the urothelium in EM is also unclear. The biopsy sites were not uniformly selected to avoid bladder hemorrhage, which could also lead to differences in the EM findings. In addition, tissue processing for EM also might impact on the EM findings, particularly if urothelium junctional proteins are affected in IC/BPS. Further study with a larger case number and immunochemical staining for EM are necessary to explore the role of umbrella cells in the pathogenesis of IC/BPS.

In conclusion, our results revealed decreased urothelial cell layers and loss of umbrella cell in the patients with IC/BPS. Umbrella cell pleomorphism and reduced microplicae of the cell membrane on SEM were also noted. The severity of umbrella cell loss and the decrease in the microplicae of the cell membrane on EM were associated with clinical symptom severity. This EM study suggests urothelium defects, especially in umbrella cells, may play an important role in the pathogenesis of IC/BPS.
